# Single-vesicle intensity and colocalization fluorescence microscopy to study lipid vesicle fusion, fission, and lipid exchange

**DOI:** 10.3389/fnmol.2022.1007699

**Published:** 2022-12-01

**Authors:** Alexandra Andersson, Marco Fornasier, Katarzyna Makasewicz, Tinna Pálmadóttir, Sara Linse, Emma Sparr, Peter Jönsson

**Affiliations:** Department of Chemistry, Lund University, Lund, Sweden

**Keywords:** vesicle fission and fusion, lipid exchange, alpha synuclein, single-vesicle imaging, fluorescence microscopy

## Abstract

Interactions of lipid vesicles play important roles in a large variety of functions and dysfunctions in the human body. Vital for several biochemical functions is the interaction between monomeric proteins and lipid membranes, and the induced phenomena such as fusion between vesicles and cell membranes, lipid exchange between the membranes, or vesicle fission. Identification of single events and their frequency of occurrence would provide valuable information about protein-lipid interactions in both healthy and degenerative pathways. In this work, we present a single-vesicle intensity and colocalization fluorescence microscopy assay with a custom-written MATLAB analysis program. The assay can be used to study lipid exchange as well as vesicle fusion and fission between two vesicle populations labeled with different fluorescent dyes. Vesicles from the two populations are first mixed and docked to a glass surface. The sample is then simultaneously imaged using two separate wavelength channels monitoring intensity changes and colocalization of vesicles from the two populations. The monomeric pre-synaptic protein α-synuclein (α-syn) and small unilamellar vesicles consisting of 1,2-dioleoyl-sn-glycero-3-phosphocholine (DOPC), 1,2-dioleoyl-sn-glycero-3-phospho-L-serine, (DOPS), and monosialotetrahexosylganglioside (GM1) were used as a model system to evaluate the method. From our analysis, neither α-syn induced fusion nor lipid exchange was observed for vesicles consisting of DOPC:DOPS (7:3). However, including 10% GM1 in the vesicles resulted in a 91% increase of the number of vesicles within 10 min, combined with a 57% decrease in the average fluorescence intensity per vesicle, indicating that approximately half of the vesicles underwent fission. The method facilitates the study of lipid vesicle fusion, fission, and lipid exchange under controlled conditions. It also allows these events to be studied for systems with more complex composition including exosomes and lipid-based drug carriers, to enable a better understanding of their physicochemical properties.

## Introduction

Fusion and fission of lipid membranes play important roles in a variety of functions and dysfunctions in the human body (Février and Raposo, 2004; Raposo and Stoorvogel, 2013), such as cytokinesis (Finger and White, 2002), mitochondrial networking (Furt and Moreau, 2009), and endo-and exocytosis of the neuron membrane neurotransmitters at the pre-synapses (Südhof, 1995). These phenomena are often enhanced by protein complexes such as the SNARE complex that mediates lipid membrane fusion (Fix et al., 2004; Han et al., 2017), or monomeric proteins such as G proteins believed to assist in the docking process (Vitale et al., 2000). Lipid molecules can be exchanged between different membranes or lipid vesicles, a process that can be aided by lipid transfer proteins (Jähnig, 1984; Kinoshita et al., 1993; Lipp et al., 2020; Peretti et al., 2020). Detection and quantification of vesicle fusion, fission, and lipid exchange with high sensitivity is therefore of great importance to better understand under which conditions these events take place and how they are influenced by protein-lipid interactions in both healthy and degenerative pathways *in vivo*. In this study, we present a single-vesicle intensity and colocalization fluorescence microscopy assay to characterize vesicle fusion, fission, and lipid exchange between discrete vesicles.

Fusion and fission of lipid vesicles can be detected by changes in vesicle and population size, which traditionally have mainly been studied using bulk techniques. This includes measuring changes in the average vesicle size using dynamic light scattering (DLS; Hallett et al., 1991; Pencer and Hallett, 2003) and fluorescence resonance energy transfer (FRET) spectroscopy (Thorsteinsson et al., 2020). Changes in vesicle size and lipid exchange have also been studied using fluorescence cross-correlation spectroscopy (FCCS) that detects both temporal and spatial cross-correlation between two channels using two differently labeled sub-populations of vesicles (Bacia et al., 2002; Cypionka et al., 2009; Makasewicz et al., 2022). However, DLS only gives the average vesicle size and does not provide information about lipid exchange, and FRET spectroscopy is not able to detect fission events. FCCS can give information about vesicle fusion, fission, and lipid exchange (Makasewicz et al., 2022); however, correct quantification of these events typically requires elaborate fitting processes. Moreover, these and other bulk-based methods only give an average value across the population, and it is thus problematic to investigate heterogeneous samples and to accurately quantify the degree of vesicle changes. The shortcomings with bulk methods can be overcome by using a single particle method to study fusion, fission, and lipid exchange on individual vesicles. This includes various imaging methods such as cryo-TEM that has previously been used to study size and morphology of single vesicles with high spatial resolution (Yuana et al., 2013; Arraud et al., 2014; Höög and Lötvall, 2015; Lou et al., 2017; Zabeo et al., 2017; Cornell et al., 2020; Emelyanov et al., 2020; Heberle et al., 2020). However, measuring lipid exchange between otherwise similarly sized vesicles is not possible, and with cryo-TEM traditionally showing a projected two-dimensional image of the vesicles, this can make it nontrivial to differentiate between vesicle deformation, and fusion and fission (Fusco et al., 2016; Man et al., 2020; Makasewicz et al., 2021, 2022). It is also possible to use fluorescence microscopy in combination with fluorescently labeled vesicles to study vesicle fusion, fission, and lipid exchange. For example, confocal laser scanning microscopy (CLSM) has been used to study curvature-specific binding of the protein Gβ1γ2 to vesicles (Hatzakis et al., 2009) and total internal reflection fluorescence (TIRF) microscopy has previously been used to study single vesicle calcium-induced vesicle leakage (Flagmeier et al., 2017) and α-synuclein (α-syn) induced vesicle clustering (Lautenschläger et al., 2018). By mixing two types of vesicles with one fluorescent dye of a FRET pair each, it is possible to detect and quantify docking, lipid exchange, or vesicle fusion between single vesicles using TIRF microscopy (Bowen et al., 2004; Yoon et al., 2006; Chan et al., 2009; Diao et al., 2012). Furthermore, docking and fusion of vesicles into a supported lipid bilayer (SLB) have been studied by detecting changes in intensities of vesicles labeled with one fluorescent dye (Fix et al., 2004; Liu et al., 2005; Simonsson et al., 2010).

**Figure 1:**
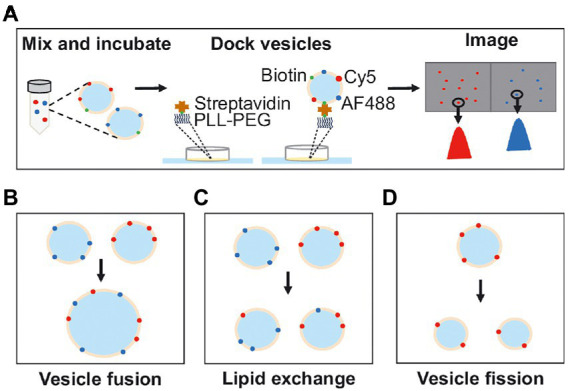
**(A)** Schematic illustration of the experimental procedure. Vesicles from two differently labeled populations are incubated together. After incubation, the vesicles are docked onto a passivated glass surface and imaged in two wavelength channels. The images are analyzed by detecting the positions, number of vesicles, and vesicle intensity. **(B)** Fusion between two differently labeled vesicles results in a reduced number of vesicles and colocalization of vesicles in the two wavelength channels with intensities similar to before vesicle mixing. **(C)** Lipid exchange results in an unchanged number of vesicles and colocalization between the vesicles in the two wavelength channels but with varying intensities compared to before vesicle mixing. **(D)** Fission, where only one vesicle population is needed, results in an increase in the number of vesicles and a decrease in the vesicle intensity.

Inspired by previous methods we developed a single-vesicle microscopy method combined with a custom-written analysis program to study fusion, fission, and lipid exchange between single vesicles. For this, two populations of vesicles, labeled with separate fluorescent dyes, are used (Figure 1A). The vesicles are mixed under the experimental conditions studied, and after mixing they are quickly diluted to single-vesicle concentrations and deposited on a microscope cover slide for observation in the two channels. Each vesicle in the two channels is detected using a custom-written MATLAB analysis program providing information about the fluorescence intensity and the position of the vesicle. After fusion, vesicles will colocalize in the two-color channels, the total number of vesicles will decrease and the fluorescence intensity per vesicle will, in each channel, remain constant or increase (Figure 1B). After lipid exchange, vesicles will again colocalize in the two channels, but the number of vesicles will remain unchanged and the fluorescence intensity per vesicle, in each channel, will decrease (Figure 1C). After fission, the vesicles will not colocalize in the two channels, the number of vesicles will increase and the fluorescence intensity per vesicle will decrease (Figure 1D). Thus, from the number of detected vesicles, the number of colocalized vesicles observed in the two channels, and the vesicle fluorescence intensities, quantitative information about vesicle fusion, fission, and lipid exchange can be obtained.

As a model system for our single-vesicle assay, we investigate the behavior of small unilamellar lipid vesicles being exposed to the amyloidogenic protein α-syn, an intrinsically disordered pre-synaptic protein mainly present in dopaminergic neurons. α-syn has been shown to be involved in several neurodegenerative diseases such as Lewy Body Dementia and Parkinson’s disease (Kim et al., 2004; Calì et al., 2014; Burré, 2015) as one of the main components of large inclusion bodies (Lewy bodies) of protein and lipids, which is the hallmark of the diseases (Fahn, 2008). The function of the protein in the synapses in a healthy neuron is not yet fully understood, but monomeric α-syn is known to interact with acidic lipids such as 1,2-dioleoyl-sn-glycero-3-phospho-L-serine (DOPS) and monosialotetrahexosylganglioside (GM1). α-syn-induced fission, disruption, fusion, or clustering may hence disturb the vesicle trafficking in the neuron and interfere with the intercellular signaling (Lee and Kim, 2017; Lewis, 2021). When bound to the membrane, the positively charged N-terminus of the protein forms an amphipathic α-helix with hydrophobic side chains preferentially embedded in the upper acyl layer and the charged side chains in the lipid headgroup area (Davidson et al., 1998). Binding has been detected at physiological pH (Jo et al., 2000) but is enhanced in more acidic environment (Hellstrand et al., 2013). Since intracellular lysosomes have a pH of 5.5, it has been argued that pH variations might influence neurotransmitter release in the synaptic cleft (Ahdut-Hacohen et al., 2004). Further, an acidic pH renders the C-termini of the proteins less negatively charged and reduces the electrostatic repulsion to negatively charged lipids. The protein is believed to promote clustering of vesicles as an initial step of docking and fusion (Diao et al., 2012; Fusco et al., 2016; Lautenschläger et al., 2018; Fouke et al., 2021), and further to assist the SNARE complex in fusion of vesicles into the cell membrane (Yoon et al., 2006; Diao et al., 2012). It has also been found that inclusion of 1,2-Dioleyol-sn-glycero-3-phospho-rac-(1-glycerol) (DOPG) in vesicles leads to α-syn-induced membrane disruption (Hannestad et al., 2020), and that co-aggregation of α-syn and GM1-containing vesicles results in a reduced vesicle size, indicting fission (Gaspar et al., 2021), likely due to the large headgroup and short tail of the lipid, which allows the protein to penetrate deeper into the membrane upon association. Using our single-vesicle assay we can here confirm these views and show that incubation of α-syn with DOPC:DOPS (7:3) does not induce fusion, fission or lipid exchange over 48 h. However, analyzing vesicles consisting of 10% of GM1 incubated with α-syn with our assay, showed significant vesicle fission already within 10 min.

## Materials and methods

### Lipids and α-syn

The following lipids were used: 1-palmitoyl-2-oleoyl-sn-glycero-3-phosphocholine (POPC), 2-dioleoyl-sn-glycero-3-phosphocholine (DOPC), 1,2-dioleoyl-sn-glycero-3-phospho-L-serine (DOPS), and monosialotetrahexosylganglioside (GM1). All lipids were purchased from Avanti Polar Lipids and diluted in chloroform to reach a desired stock concentration of 0.1–10 mg/ml. The samples used for the experiments were (i) POPC vesicles as control, (ii) DOPC:DOPS (7:3 molar ratio) used as control and as a model system for interactions with monomeric α-syn, and (iii) DOPC:DOPS:GM1 (7:2:1 molar ratio) used as a model system for interactions with monomeric α-syn. Small unilamellar vesicles of type (ii) and (iii) in addition contained 1 mol% of the lipid 1-oleoyl-2-[12-biotinyl(aminododecanoyl)]-sn-glycero-3-phosphocholine (18:1–12:0 Biotin PC). “Blue” labeled vesicles furthermore contained 1 mol% of 1,2-dioleoyl-sn-glycero-3-phosphoethanolamine-N-(TopFlour®AF488) (18:1 PE-TopFlour®AF488) and the “red” labeled vesicles 1 mol% of 2-dioleoyl-sn-glycero-3-phosphoethanolamine-N-(Cyanine 5) (18:1 Cy5 PE). For the control measurements “red + blue” vesicles containing 0.5–1 mol% of both fluorescently labeled lipids were used. Wild-type human α-syn was expressed in *Escherichia coli* BL21 DE3 PLysS from a Pet3a plasmid carrying a synthetic gene with *E. coli*-optimized codons, and purified using heat treatment, ion exchange and gel filtration chromatography as described previously (Grey et al., 2011).

### Lipid vesicle preparation

Lipid vesicles were prepared from stocks of 0.1–10 mg/ml lipids in chloroform. The lipids were aliquoted in clean glass vials and the chloroform was evaporated by insufflating a gentle nitrogen flow. After complete removal of the organic solvent, the thin lipid film was hydrated in buffer solution to reach a final concentration of lipids of 1–4 mM. The POPC samples for the control measurements were hydrated in 10 mM 4-(2-hydroxyethyl)-1-piperazineethanesulfonic acid (HEPES) buffer from Thermo Fisher, at pH 7.4, containing 150 mM NaCl. The DOPC:DOPS and DOPC:DOPS:GM1 samples were hydrated in a 10 mM MES buffer at pH 5.5, containing 200 μM NaN₃. To obtain small unilamellar vesicles, the samples were vortexed and sonicated using a tip sonicator (VCX 130; Sonics & Materials, Inc.) in pulse mode (10 s of sonication followed by 10 s of break) at 71.5 W for 15 min of total sonication time. This resulted in 50 nm diameter vesicles (checked by Malvern DLS; data not shown).

### Surface passivation and sample preparation

All measurements were conducted in sterile 10 μl silica culture wells from Grace BioLabs attached to glass cover slides (25 mm diameter, thickness 1.5, Menzel-Gläzer) cleaned in a sulfuric acid (99.9%, Sigma-Aldrich): hydrogen peroxide (30%, Sigma-Aldrich) (3:1 v:v) solution for 30 min at 80°C. The initial control measurements with POPC vesicles were added directly to the clean slides. The remaining samples were tethered to cleaned glass slide using biotin-streptavidin linkage. Culture wells were incubated with a 0.1 mg/ml PLL20-g[3.5]-PEG(2): PLL20-g[3.5]-PEG(2)/PEG3.4-biotin (20%) (SuSoS AG, Switzerland), solution (100:1 w:w) in phosphate-buffered saline (PBS) solution at pH 6.5 for 30 min. After incubation, the wells were incubated with 0.1 mg/ml streptavidin (Thermo Fisher) in PBS at pH 6.5 for 15 min before rinsing the sample with experimental buffer. All control samples were diluted to a total lipid concentration of 50 nM, with different ratios of red and blue labeled vesicles, before imaging.

The samples incubated with α-syn were incubated at a lipid concentration between 0.5 and 2 mM and a protein concentration between 5 and 10 μM, resulting in a lipid:protein ratio (L:P) between 50 and 400. Before imaging, samples with DOPC:DOPS vesicles were diluted to a total lipid concentration of 50 nM, i.e., 25 nM red vesicles and 25 nM blue vesicles. Vesicles consisting of DOPC:DOPS:GM1 were diluted to a final concentration of 20 nM before imaging.

### Fluorescence microscopy

The microscope system consisted of a customized Nikon TE-2000 U inverted microscope combined with a Nikon Apo TIRF oil objective (*M* = 60×, NA = 1.49). Two diode lasers from the Cobolt 06.01 series, with central wavelengths of 488 nm and 638 nm, were guided into the microscope for TIRF illumination of the blue and red channels, respectively. TIRF microscopy has the advantage of reducing the signal from fluorescent molecules in the bulk solution. TIRF is for the current experiments not strictly needed since the fluorescence from vesicles in the bulk solution is very low, thus other microscopy techniques such as confocal microscopy and standard epifluorescence microscopy could also be used in the single-vesicle assay under these conditions. Fluorescence from the illuminated samples was split by a dichroic mirror, (405/488/532/635 BrightLine®, Semrock) and filtered by emission filters (FF01 512/25 and FF01 650/13 from Semrock). The two fluorescence wavelengths were separated by a Hamamatsu W-WIEW GEMINI (A12801-0) and imaged as two sub-images onto a 2048×2048 Hamamatsu ORCA-Flash4.0 V3 digital scientific CMOS camera (C13440) with a pixel size of 6.5 × 6.5 μm. All measurements were conducted using a 2×2 binning and exposure time of 100 ms.

### Analysis program

A custom-written program in MATLAB was developed to analyze the data. The program reads .tif-files with different sizes, in either one or two different wavelength channels. In case of images obtained with one wavelength channel, the program will only detect the single vesicles and return variables as intensity and size. Additionally, if the image is obtained using two wavelength channels, vesicles are colocalized between the channels. The program is available at https://github.com/jopeterLU/programs.

All data presented are calculated as mean ± SD from n = 3–5 measurements. For each measurement, vesicle data from five different images were acquired and averaged. A two-sample t-test in MATLAB was used for the statistical analysis.

#### Particle detection

Raw data are read from .tif-files into MATLAB 2020b as two-dimensional arrays and filtered by a low-pass filter. The background is subtracted by looping a rolling ball algorithm over all columns of the image to compensate for the Gaussian illumination profile of the laser beams. The background level is defined by


(1) $$\mathrm{BG}=\left[\mathrm{col}\right]-\sqrt{r^{2}-{\left(kdx\right)}^{2}}$$

where [col] is a column in the two-dimensional image, r is the radius of the rolling ball, k is the current pixel, and dx is the spacing of sample points. The background is filtered by a median filter over a 3×3-pixel area and then subtracted from the raw data image. The background-subtracted image is finally subjected to a Gaussian filter.

**Figure 2:**
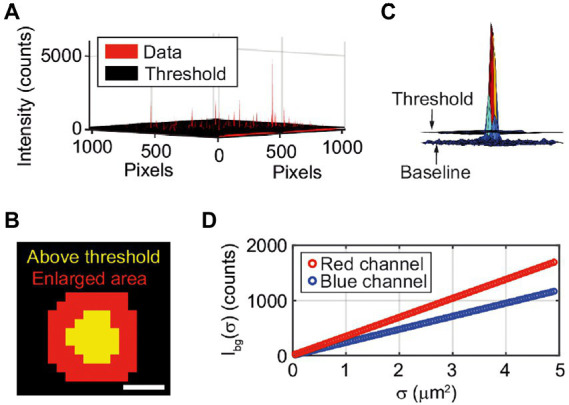
**(A)** Vesicles are detected using a 2-dimensional threshold (Eq. 2) matrix based on a user-determined SFR (here SFR = 2). All red peaks above the black threshold “floor” are detected vesicles. Note that since the vesicles are smaller than the diffraction limit of light the measured spot size in the fluorescence microscopy images does not correspond to their actual size. **(B)** The area that is detected as a pixel is enlarged by a 5 × 5 matrix. The scale bar is 1 μm. **(C)** Intensity from a single vesicle, threshold and baseline. **(D)** The background intensity (*I*_BG_) dependence on the spot size (σ) for the blue and red channel.

Single vesicles in the image are detected by creating a binary mask of the pixels that passes an intensity threshold depending on a user-determined signal-to-fluctuation ratio, SFR. The SFR is set by the user at the beginning when using the program and can be adjusted for every image if needed. The fluctuation level is pixel specific and is defined as twice the difference between the median, *I*_median_, and minimum, *I*_min_, intensity row vectors of the two-dimensional image. The intensity threshold is defined as

(2) $$I{\left(r,:\right)}_{\mathrm{th},\mathrm{pos}}=\mathrm{SFR}*2\left(I_{\mathrm{median}}\left(r,:\right)-I_{\min}\left(r,:\right)\right)$$

and is hence specific for every pixel, depending on the two-dimensional fluctuation level. This eliminates further issues with uneven background intensities due to inhomogeneous background illumination. The pixels that pass the threshold, i.e. a “spot”, represent a single vesicle (Figure 2A). Every spot is enlarged by a 5×5 pixel window, corresponding to an enlargement of a 2-pixel area around the detected spot (Figure 2B). The enlargement is performed to include the edges of the spot that might not pass the threshold (Figure 2C). Further, the intensity of the enlarged area of a spot is integrated to obtain the integrated intensity, *I*_int_


(3) $$I_{\mathrm{int}}=\overset{n}{\underset{i=1}{\sum }}I_{\mathrm{pixel}}\left(i\right)$$

Since the same enlargement is applied to all spots, the additive effect is equal for all spots. If no enlargement is done, failing to include the edges of the spot would lead to greater differences in *I*_int_ due to the spread in the intensity distribution of the vesicle population.

The background intensity is calculated for each image by randomly choosing hundred spots from places where no vesicles are detected and integrate the intensities. The sizes of the spots vary from 10 to 100 pixels. The randomly chosen data is used to make a fit of the background intensity dependence on the spot size, *σ*, (Figure 2D). A new integrated intensity, *I*_tot_, normalized to the background intensity of a spot of corresponding size, can now be defined as:


(4) $$I_{\mathrm{tot}}\left(\sigma \right)=\left(I_{\mathrm{int}}-I_{\mathrm{BG}}\left(\sigma \right)\right)/I_{\mathrm{BG}}\left(\sigma \right)$$

The corresponding peak intensity is


(5) $$I_{\mathrm{peak}}=\left(I_{\max}-I_{\mathrm{BG}}\left(1\;\mathrm{pixel}\right)\right)/I_{\mathrm{BG}}\left(1\;\mathrm{pixel}\right)$$

where *I*_max_ is the maximum intensity of the detected spot. *I*_tot_, the total intensity of the spot normalized to the background, is used to detect lipid exchange. During measurements, *I*_peak_, the maximum intensity of the spot normalized to the background, is tracked as well to relate peak intensity to integrated intensity, and to compare size distributions of these two intensity variables.

#### Colocalizing particles in two channels

**Figure 3:**
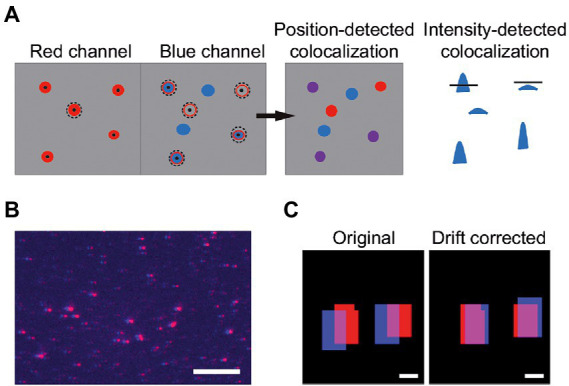
**(A)** Vesicles can be colocalized by comparing positions. Here, an area around the center positions of detected vesicles in the first channel is used to search for center positions in the second channel (position-detected colocalization). Colocalization can also be detected by comparing the integrated intensities over the same areas in the two channels. The mask from one channel is applied to the second cannel and I_tot_ is calculated. The intensities that pass a SFR-based threshold (Eq. 6) are colocalized (intensity-detected colocalization). **(B)** Image of an overlay of the red and blue channels of a sample with 100% theoretical colocalization. All red spots are slightly shifted compared to the blue spots, indicating that drift correction is needed. The scale bar is 10 μm. **(C)** Binary image of two detected spots before (left) and after (right) drift correction. The scale bars are 1 μm.

In the case of a two-channel image, the procedure described above is executed for each channel separately. When the spots in the two channels are detected, the program searches for colocalization of vesicles between the two sub-images. Colocalization can be detected by two different methods. The first colocalization method compares the positions of the spots in both sub-images and see if there is a vesicle with a center position within a user-determined area in the second channel (Figure 3A). This method will further on be referred to as position-detected colocalization. The second colocalization method applies the mask from one channel on the second channel and calculates *I*_tot_ in the second channel. If this integrated intensity is above a threshold set by


(6) $$I_{\mathrm{th},\mathrm{int}}\left(\sigma \right)=\mathrm{SFR}*I_{\mathrm{BG}}\left(\sigma \right)$$

the vesicles are considered colocalized. This method will further on be referred to as intensity-detected colocalization (Figure 3A). To compensate for differences in positional alignment and chromatic effects between the two channels the user has the possibility to “drift correct” the images (Figure 3B C). To do this, the two sub-images are cross-correlated. The drift offset between the sub-images is obtained by finding the maxima in the correlation matrix. If the user does not want to drift correct, the program gives the alternative to shift the channels before starting the analysis. The drift of the detection system must then be known beforehand. This can be done using a reference colocalized sample and analyze the images with drift correction. The offset can then be applied to the rest of the images that are analyzed without drift correction.

When the program has analyzed colocalization of the first image in the stack, a composite of the binary masks for the two sub-images are shown with the colocalized spots circled in white. Spots are plotted either red, for the left channel, or blue for the right channel. The user then has the choice to continue or cancel the analysis. If the user decides to continue, this can be done either interactively as with the first image or non-interactively, meaning that the image with detected spots or the composite of colocalized spots will not be shown, and that the program will analyze the remaining images without presenting the user with the possibility to change parameter values.

## Results

### Single-vesicle detection and characterization in one channel

**Figure 4:**
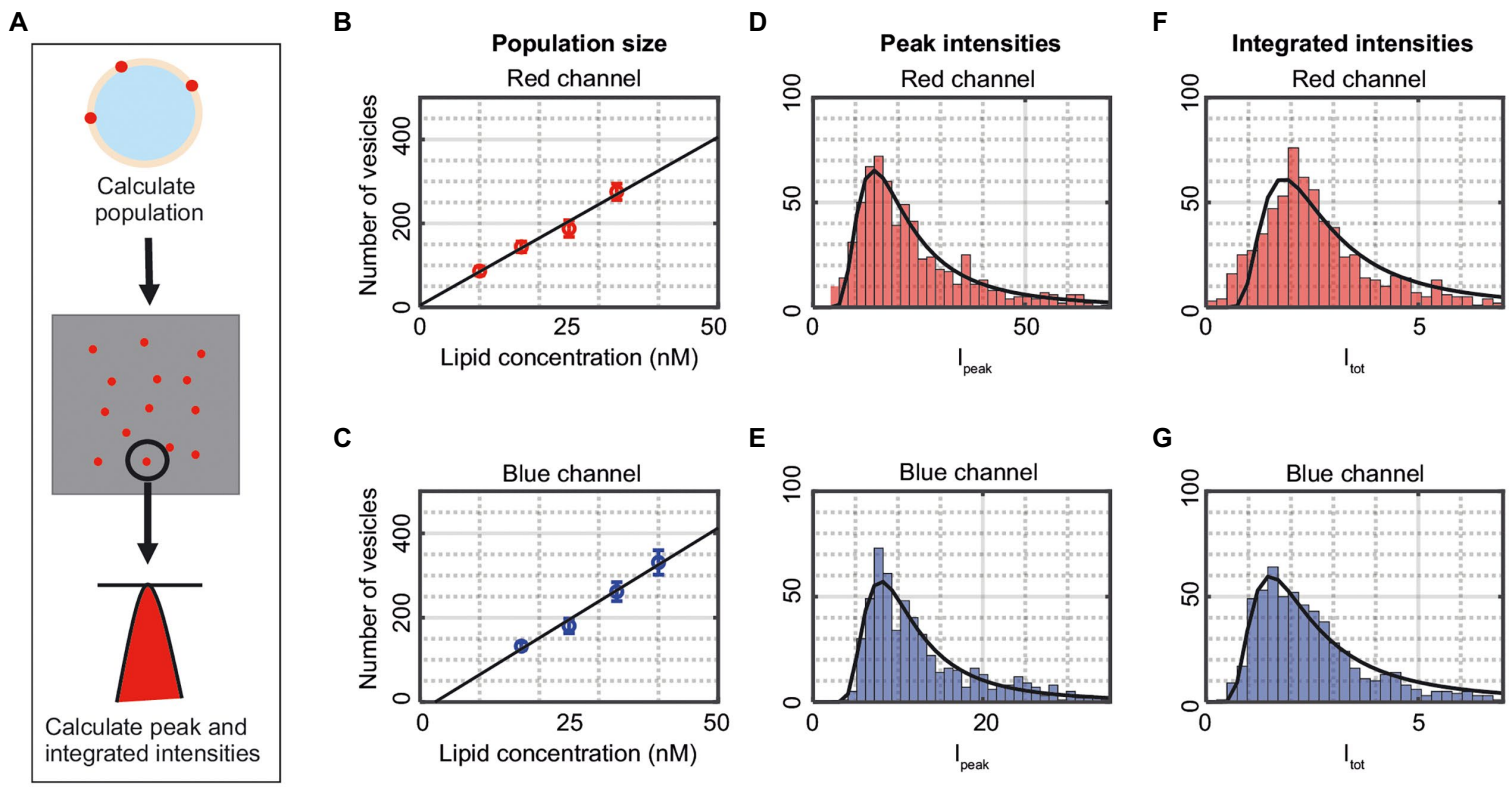
**(A)** Illustration of the detection process. The vesicles are shown as spots in the two-dimensional image. The number of vesicles is counted, and the peak intensity (I_peak_) as well as the integrated intensity (I_tot_) of the individual vesicles are calculated. **(B,C)** The number of detected vesicles in the channels as a function of added lipid concentration. The data correspond to mean ± SD from n = 3–5 measurements. The solid lines are linear fits to the data. **(D,E)** Peak intensities, I_peak_ (Eq. 5), of vesicles in the red and blue channel from one representative measurement. The solid lines are fits to a Weibull distribution. **(F,G)** Integrated intensities, I_tot_ (Eq. 4), of vesicles in the red and blue channel from one representative measurement. The solid lines are fits to a Weibull distribution.

The ability to detect vesicle fusion, fission, and lipid exchange is dependent on the ability to correctly detecting single-vesicle intensities and positions. For this purpose, vesicles labeled either with AF488 (blue) or Cy5 (red) were used (Figure 4A). The number of adsorbed vesicles on the glass slide was found to scale linearly with the concentration of added vesicles (Figures 4B,C) and at a concentration of 25 nM lipids, the number of adsorbed vesicles was just below 200 for both the blue and red channel. There is typically a spread in the peak fluorescence intensity, *I*_peak_ (Eq. 5), from a vesicle (Figures 4D,E), partly due to differences in illumination across the sample, an issue that can be reduced by image cropping and analysis of vesicles from only the part of the image where the illumination is most flat. However, the main reason for the spread in intensity is due to an inherent spread in the vesicle size within the vesicle population. Figures 4F,G show the normalized integrated intensity, *I*_tot_ (Eq. 4), from each detected vesicle, which has a similar spread as the peak intensities. Both the *I*_peak_ and *I*_tot_ distributions could be fitted by a Weibull distribution since these intensities, for small vesicles, scale approximately linearly with vesicle size, in accordance with Fang et al. (1993). The fits to *I*_peak_ (*I*_tot_) gave a population maximum of 8.2 (1.8) and 14.4 (1.5) for the blue and red channel, respectively. Both intensity variables have similar accuracy, but for simplicity only *I*_tot_ is presented in the following results.

### Vesicle detection and colocalization in two channels

**Figure 5:**
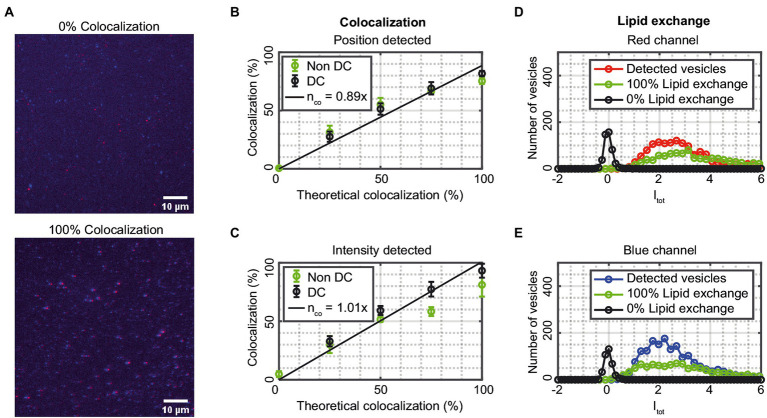
**(A)** Fluorescence images of two samples of vesicles, either all vesicles are blue or red (0% colocalization) or all vesicles are both blue and red (100% colocalization). The red and blue channels are merged, and the scale bars are 10 μm. **(B,C)** The measured colocalization as a function of theoretical colocalization for data that is not drift corrected (green) and data that is drift corrected (black). The data corresponds to mean ± SD from n = 3–5 measurements. The solid lines are linear fits to the drift corrected data. **(D,E)** The integrated intensity I_tot_ (Eq. 4) distribution for the vesicle population collected from 1 to 3 measurements. Detected vesicles correspond to vesicles that are detected in the red (blue) channel and 0 and 100% lipid exchange refers to values when the mask from the blue (red) channel is applied to the red (blue) channel for samples with 0 and 100% lipid exchange.

The accuracy of the colocalization by the single-vesicle assay was assessed by measuring on vesicles having blue, red, or blue + red fluorophores in different molar ratios (Figure 5A). The ratios were chosen to correspond to 0, 25, 50, 75, and 100% vesicles containing both dyes and thus mimicking fusion events. Figures 5B,C show the percentage of colocalized vesicles detected compared to the theoretical values from the mixed samples for position-detected and intensity-detected colocalization, respectively. The solid lines are linear fits of the drift-corrected data (25–100% colocalization) with slopes of 0.89 and 1.01 for position-detected and intensity-detected colocalization, respectively. Both colocalization schemes thus agree reasonably well with the theoretical values, although with a slight drop in colocalization for the 100% sample, and with better agreement with the theoretical values for the intensity-detected colocalization. A reason for this is that intensity-detected colocalization is better at detecting small or unfocused vesicles from the data that the position-detected colocalization could miss. Thus, if the data indicates sufficiently high fusion (more than 25% colocalization), the data is more accurately analyzed by intensity-detected colocalization. The experimental values of the 0% colocalized, not drift corrected, samples have a mean value of 0.53% using position-detected and 4.81% when using intensity-detected colocalization. Thus, for samples with low colocalization, position-detected colocalization is preferable whereas intensity-detected colocalization tends to find “false” vesicles from the background. Using automatic drift correction for samples with 25% or higher theoretical colocalization leads to more colocalized vesicles and values close to the theoretical ones (Figures 5B,C). Thus, automatic drift correction is advantageous on colocalized samples, but only works when there are colocalized vesicles in the image; otherwise, there will be false colocalization that results in a higher colocalization in the sample.

Figures 5D,E show the normalized integrated intensity *I*_tot_ (Eq. 4) for detected vesicles in the red (blue) channel when the positions obtained in the blue (red) channel are being used as “mask.” This was done for samples with 0% theoretical lipid exchange (no vesicles are both blue and red) and 100% theoretical lipid exchange (all vesicles are both blue and red). “Detected vesicles” refers to vesicles that are detected in the red (blue) channel and 0 and 100% exchange refers to when the mask from the blue (red) channel is applied to the red (blue) channel for samples with 0 and 100% lipid exchange. The results in Figures 5D,E validate the colocalization analysis as well as provide a method to quantify lipid exchange, and fusion by comparing the obtained values with the corresponding <*I*_tot_ > for the “Detected vesicles” in that channel. From Figure 5D,E, the 0% lipid exchange sample has an <*I*_tot_ > of 0.05 ± 0.03 and 0.09 ± 0.04 for the red and blue channel, respectively, which can be compared to <*I*_tot_ > for the detected vesicles of 3.60 ± 0.64 and 2.32 ± 0.93, corresponding to a lipid exchange of 1 ± 1% and 4 ± 2% for the red and blue channel, respectively. This sets a limit for the sensitivity of detecting lipid exchange in the two channels. For the 100% lipid exchange sample the corresponding <*I*_tot_ > values are 3.65 ± 1.23 and 2.35 ± 0.42 for the red and blue channel, respectively, which are similar to the values for the detected vesicles, corresponding to a lipid exchange of 101 ± 39% and 101 ± 44% for the red and blue channel, respectively. There was no statistically significant difference between the detected vesicles and the 100% colocalized sample for neither channel. However, there was a statistically significant difference between the detected vesicles and the 0% colocalized sample (*p* < 0.01 for both channels).

### α-syn does not induce vesicle fusion or lipid exchange in DOPC:DOPS vesicles

**Figure 6:**
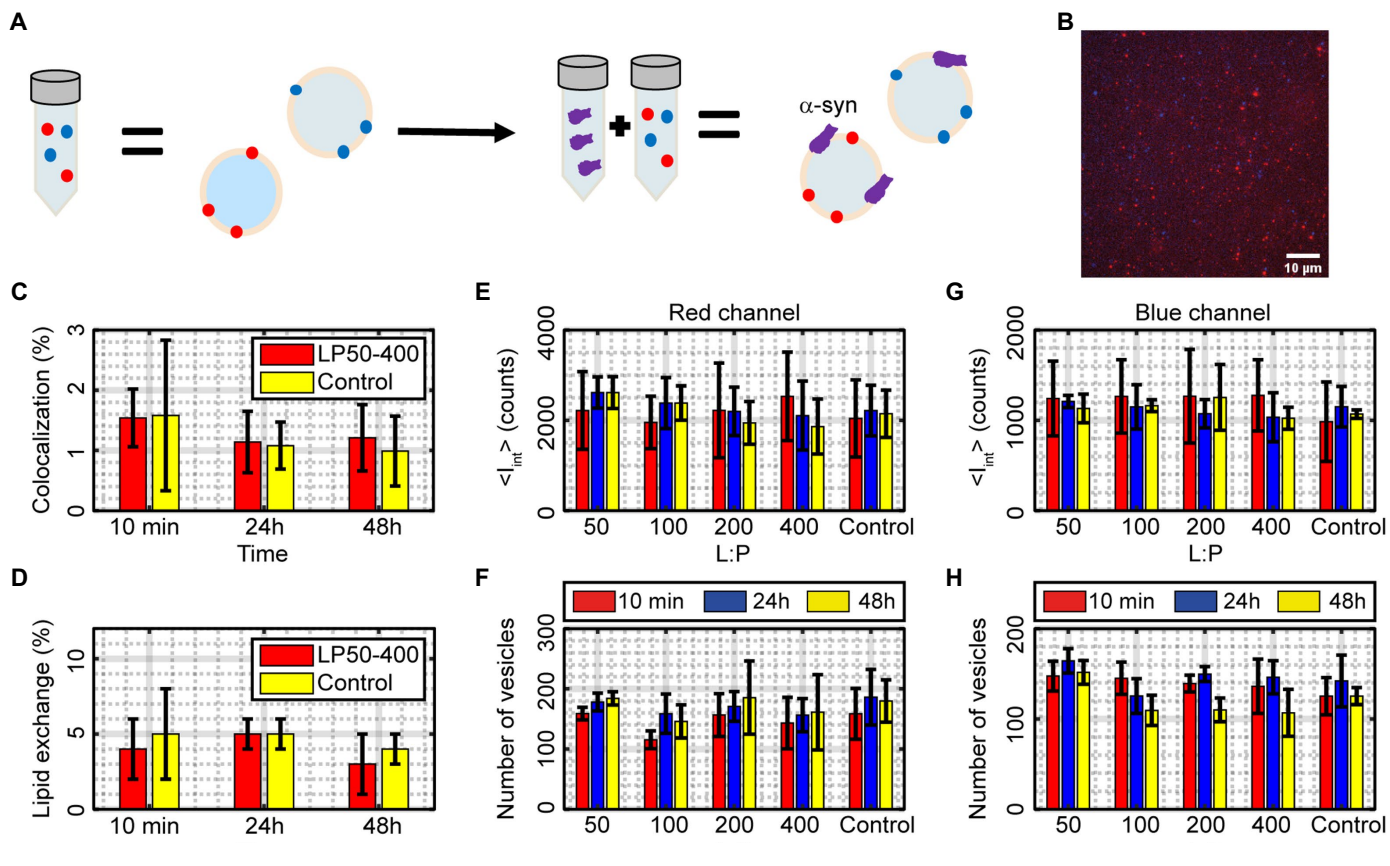
**(A)** Illustration of the mixing of α-syn with vesicles and the resulting outcome. **(B)** A fluorescence image of blue and red vesicles mixed with α-syn and docked to a glass slide for single-vesicle analysis. The image is an overlay of the two wavelength channels and the scale bar is 10 μm. **(C,D)** Measured percentage of colocalized vesicles and amount of lipid exchange after incubating DOPC:DOPS vesicles with α-syn compared to vesicles without α-syn (Control). The results are averages over all L:P ranges. The data correspond to mean ± SD from n = 3 measurements. **(E–H)** The number of vesicles and average integrated intensity <*I*_int_ > (Eq. 3) in the red and blue channel for different L:P ratios and incubation times compared to the control sample without α-syn. The data correspond to mean ± SD from *n* = 3 measurements.

α-syn was incubated with DOPC:DOPS (7:3) vesicles at different lipid-to-protein ratios for up to 48 h after which the samples were analyzed using the single-vesicle intensity and colocalization fluorescence microscopy assay (Figures 6A,B). Samples incubated with α-syn had an average of 1.3 ± 0.2% position-detected colocalization over all time points, compared to control vesicles that had 1.2 ± 0.3% colocalization (Figure 6C), which is equivalent to no detectable fusion within the experimental accuracy. The <*I*_tot_> value averaged over all time points for vesicles incubated with α-syn was for the red channel 0.08 ± 0.03 and for the blue channel 0.06 ± 0.03. Both these values are within error the same as the corresponding values for vesicles without α-syn (Control) that had an <*I*_tot_> of 0.09 ± 0.02 and 0.05 ± 0.02 for the red and blue channel, respectively. To get a measure of lipid exchange, these values can be scaled to the corresponding values for “detected vesicles,” which were 1.66 ± 0.28 and 1.44 ± 0.23 for the red and blue channel, respectively, resulting in an average lipid exchange for the two channels of 4 ± 2% in absence of α-syn and 5 ± 3% in presence of α-syn (Figure 6D). There was no statistically significant difference between vesicles in absence and presence of α-syn. Also, the number of vesicles and <*I*_int_> (Eq. 3) behaved similarly to the control vesicles and did not change significantly over 48 h (Figures 6E–H). Altogether, this indicates that α-syn does not induce any vesicle fusion, fission or lipid exchange within 48 h for vesicles consisting of DOPC:DOPS.

### α-syn induces vesicle fission in GM1-containing vesicles

**Figure 7:**
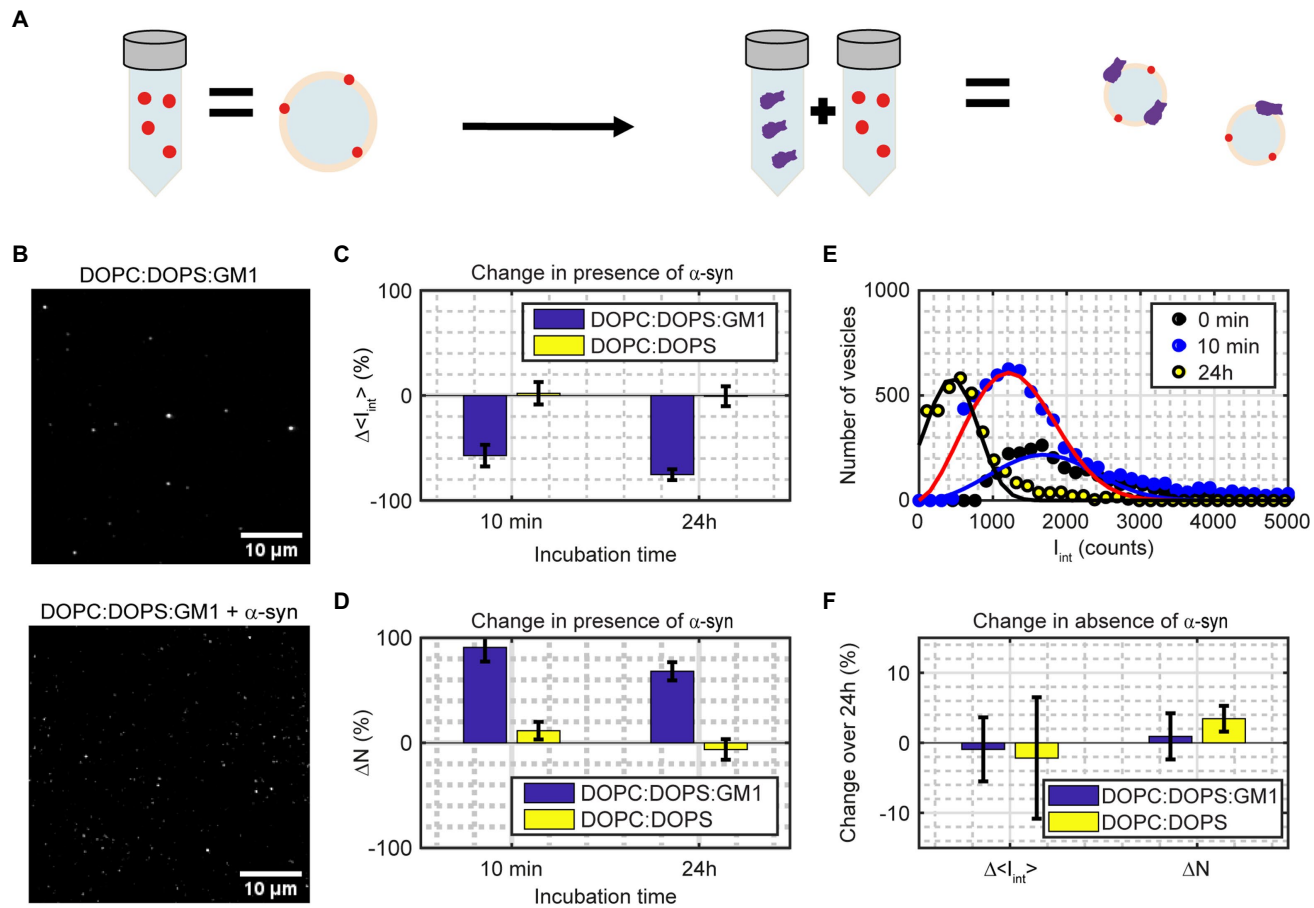
**(A)** Illustration of the measurement. Mixing a sample of fluorescently labeled vesicles containing 10 mol% GM1 with α-syn results in more vesicles but with lower intensity per vesicle. **(B)** Image of samples in absence (top) and presence (bottom) of α-syn. The scale bars are 10 μm. **(C,D)** Relative changes in integrated intensity (Δ < *I*_int_>; Eq. 3) and number of vesicles (ΔN) for vesicles containing DOPC:DOPS:GM1 (7:2:1) and DOPC:DOPS (7:3) incubated with α-syn compared to vesicles without α-syn. The data correspond to mean ± SD from n = 4 measurements. **(E)** *I*_int_ (Eq. 3) population distribution from one representative measurement for DOPC:DOPS:GM1 vesicles either without α-syn incubation (0 min; black circles), 10 min α-syn incubation (blue circles) or 24 h α-syn incubation (yellow circles). The solid lines are fits to a Weibull distribution. **(F)** Change in integrated intensity (Δ < *I*_int_>) and number of vesicles (ΔN) for DOPC:DOPS:GM1 and DOPC:DOPS vesicles over 24 h in absence of α-syn. The data correspond to mean ± SD from n = 3–4 measurements.

To investigate fission with our single-vesicle assay, we added GM1 to the vesicles at a 10 mol% ratio. To detect fission, the fluorescence intensity and number of vesicles were analyzed for red labeled vesicles after being incubated with α-syn at a L:P ratio 200:1 for different times (Figures 7A,B). The inclusion of the lipid GM1 in the lipid vesicles and the addition of α-syn resulted in an increase in the number of vesicles combined with a decrease in the integrated intensity per vesicle already after 10 min (Figures 7C,D). The number of detected vesicles, *N*, increased by 91 ± 13% and the integrated fluorescence intensity per vesicle, < *I*_int_>, decreased by 57 ± 10% after 10 min of incubation with α-syn (Figures 7C,D), which is statistically significant (*p* < 0.002). Almost all vesicles had divided into smaller vesicles, but most of these vesicles were still large enough to be detected. However, the total fluorescence signal from the sample, *N* × < *I*_int_>, had decreased slightly compared to the control sample without α-syn (18 ± 23% lower) indicating that some fission, or fragmentation, into vesicles below our detection limit was taking place. There was no statistically significant change in vesicle intensity (2 ± 11%) or in population size (12 ± 8%) when the vesicles only contained DOPC:DOPS. After 24 h, the GM1-containing vesicles showed a 68 ± 9% increase in number of vesicles combined with a 75 ± 5% decrease in average intensity (Figures 7C,D), compared to the control vesicles without α-syn, which is statistically significant (*p* < 0.001) The sample had a 58 ± 10% drop in the total fluorescence signal compared to the control DOPC:DOPS:GM1 sample without α-syn after 24 h. Thus, most of the vesicles were now smaller than the detection limit. That vesicles were not detected in this case can also be observed from Figure 7E where the intensity histograms are shifted towards smaller intensity values, and after 24 h a substantial part of the left tail of the population is below the detection limit. There was again no statistically significant change in population size (6 ± 10%) or in vesicle intensity (−1 ± 9%) when the vesicles only contained DOPC:DOPS, indicating that no fission had occurred in this case. Furthermore, without α-syn, both the DOPC:DOPS and the DOPC:DOPS:GM1 vesicles appeared stable over 24 h, showing no change in intensity or number of vesicles (Figure 7F).

## Discussion

The single-vesicle intensity and colocalization fluorescence microscopy assay described here, in combination with the MATLAB analysis program, was developed to provide an interactive tool suitable for fluorescence microscopy image analysis and detection of vesicle fusion, fission, and lipid exchange. The control measurements using red, blue, and red+blue labeled vesicles with known composition showed that the method can detect colocalization and quantify differences in vesicle intensities and number of vesicles with high precision in order to characterize these events. The assay was in addition used to study how α-syn influences different lipid vesicles to better understand the physicochemical mechanism of α-syn interacting with lipid membranes.

From the analysis of DOPC:DOPS vesicles mixed with α-syn, it was concluded that α-syn does not induce detectable vesicle fusion, fission or lipid exchange between DOPC:DOPS (7:3) vesicles within 48 h. This agrees with recent conclusions by Makasewicz et al., that shape deformations of DOPE:DOPC:DOPS (5:3:2) vesicles in presence of α-syn detected by cryo-TEM (Fusco et al., 2016; Man et al., 2020), would be mainly deformation of the vesicles upon binding of the protein, without any changes to the lipid content of the vesicles (Makasewicz et al., 2022). If the vesicles contain GM1, it was found that the addition of α-syn results in a rapid increase of the number of vesicles in the sample, in agreement with previous results obtained by cryo-TEM (Gaspar et al., 2021). After 10 min, the sample contained almost twice as many vesicles as prior to α-syn addition and the average intensity per vesicle approximately halved, indicating that most of the vesicles in the sample had undergone fission once. In the absence of α-syn, the vesicle intensities remained unchanged, thus binding of α-syn to GM1-containing vesicles facilitates fission. One possible mechanism is that the large head group and the short tail of GM1 allow the protein to penetrate deeper into the membrane upon association. Altogether, this indicates that although α-syn could assist in the docking process during exo-and endocytosis in the neuron, it would likely not induce fusion or lipid exchange on its own. However, α-syn can induce vesicle fission and thereby disturb the vesicle trafficking in the neuron and interfere with the intercellular signaling.

After 24 h incubation of a mixture of GM1-containing vesicle and α-syn the most prevalent vesicle size was 4 times smaller than in the control samples without protein (Figure 7E). In addition, 58% of the vesicles were now so small that they were below the detection limit. It is thus possible from the population information to compensate for vesicles too small to be detected, whereas this is more complicated and generally requires more elaborate fitting using bulk methods. On the other hand, bulk methods such as DLS and FCCS have the advantage that no docking of the vesicles to a surface is needed. Although the results in this study are in agreement with the results obtained from bulk techniques, care must be taken when docking vesicles to the glass cover slide to avoid unspecific interactions. In addition, label-free techniques such as DLS and cryo-TEM have the advantage that no addition of fluorophores to the studied vesicles is needed to quantify changes in vesicle size. Even though the density of the fluorescent lipid in our single-vesicle assay is low (≤1 mol%) care should be taken to ensure that the fluorescent lipid does not alter the behavior of the vesicles. Data of the actual size of the vesicles can be obtained using DLS and cryo-TEM, the latter also yielding information on the size of individual vesicles although deformations of the vesicles can compromise the accuracy of this approach. It is also possible to convert the measured fluorescence intensity using our single-vesicle assay into actual vesicle size by first measuring the fluorescence intensity from one fluorescent lipid and use this to obtain the number of fluorescent lipids per vesicle which in turn can be used to estimate the vesicle size. Alternatively, fluorescent calibration samples of known size can be used to convert between fluorescence intensity and actual vesicle size (Kunding et al., 2008). Another approach to get actual vesicle sizes would be to use super-resolution fluorescence microscopy techniques that overcome the diffraction limit (Sezgin, 2017).

The sensitivity of the assay is partly limited by the homogeneity of the passivated surface and the vesicle sample. An inhomogeneous surface will result in a higher or lower number of vesicles being tethered to different areas of the surface. A deviating number of vesicles in the image can be misinterpreted as fusion or fission. The impact of this issue was minimized by always imaging each sample on at least five different areas and taking a mean value of these five images. Further, the effect of docking vesicles by biotin-streptavidin linkage on a passivated surface compared to docking vesicles on a plain glass surface using a salt-containing buffer was investigated. Ideally, properly cleaned glass slides will provide a homogeneous docking surface, but interactions between lipid bilayers or lipid bilayers and proteins are often driven by electrostatic interactions and will hence be affected by salt. This is the case for interactions between α-syn and lipid bilayers. However, both tethering the vesicles on a passivated surface in MES buffer, pH 5.5, without any extra salt, or on glass slides in HEPES buffer, pH 7.4 containing 150 mM NaCl, was found to produce similar results for the studied system. Furthermore, the homogeneity of the vesicle sample limits the sensitivity of the intensity distribution. A polydisperse sample will be more difficult to detect since small or weekly fluorescent vesicles can be dismissed during the image analysis. Combined tracking of population and intensity changes is hence crucial to assure correct detection of fission and fusion events. The minimum size and intensity depend on the noise level of the detector and on the amount of dye that is incorporated in the vesicles, which must be adjusted with respect to the stability of the lipid membrane and the saturation intensity of the detector to obtain optimal results. Hence, the detection limits of the specific system can be optimized by changing illumination intensities, using detecting sensors with lower dark current values, and changing the amount of fluorophores in the vesicles.

Automatic drift correction of the positions in the two channels can be used to improve colocalization. However, the drift correction of the wavelength channels might result in falsely colocalized vesicles if there is a low amount of colocalization in the sample. For example, for the 0% theoretical colocalization samples in Figures 5B,C the measured colocalization when automatic drift correction was used was 3.5 and 6.4% for position-detected and intensity-detected colocalization, respectively. If there is a drift between images for a sample with low colocalization then an option is to find the drift offset of the image using a reference sample of 100% colocalized vesicles, and then use that offset in the colocalization analysis without drift correcting the images. Which method that should be used (position-detected or intensity-detected) depends on the sample.

In summary, the single-vesicle intensity and colocalization fluorescence microscopy assay can detect and quantify vesicle fusion, fission, and lipid exchange events within a vesicle population based on the colocalization and characterization of vesicles with two different fluorescent dyes. For the analysis of the images, an open-source MATLAB program was developed, which can also compensate for imperfections in the imaging process. We illustrated the potential of our method by studying the influence of α-syn on lipid vesicle morphology in terms of fusion and fission events as well as lipid exchange. Although the added protein in this study was unlabeled, the method can be easily adopted to include fluorescently labeled proteins and thereby investigate the impact of absorbed amount of protein per vesicle on parameters such as lipid vesicle size and mixed content. Further, the fusion of vesicles to a supported lipid bilayer under different solution conditions can be studied by labeling the initial bilayer and the fusing vesicles with different fluorophores. It is also possible to combine the method with microfluidic handling to increase the time resolution of the experiments and for automating measurements over longer time scales. In addition, by measuring the FRET signal between the blue and red channels, it would be possible to also separate between vesicle docking (low FRET) and vesicle fusion (high FRET) events. Overall, the single-vesicle fluorescence intensity and colocalization microscopy assay opens up for detailed studies of vesicle fusion, fission, and lipid exchange and how this is influenced by different parameters.

## Data availability statement

The raw data supporting the conclusions of this article will be made available by the authors upon request, without undue reservation.

## Author contributions

AA, MF, TP, SL, and PJ: conceptualization. AA: formal analysis and investigation. PJ, MF, KM, ES, and SL: resources and supervision. AA, MF, and PJ: writing—original draft and visualization. PJ: project administration and funding acquisition. All authors contributed to the article and approved the submitted version.

## Funding

This work was supported by the European Research Council (ERC) under the European Union’s Horizon 2020 research and innovation program (grant agreement no. 757797), the Knut and Alice Wallenberg Foundation (grant number: 2016.0074), and the Swedish Research Council (grant number: 2018–03872).

## Conflict of interest

The authors declare that the research was conducted in the absence of any commercial or financial relationships that could be construed as a potential conflict of interest.

## Publisher’s note

All claims expressed in this article are solely those of the authors and do not necessarily represent those of their affiliated organizations, or those of the publisher, the editors and the reviewers. Any product that may be evaluated in this article, or claim that may be made by its manufacturer, is not guaranteed or endorsed by the publisher.

## References

[ref1] Ahdut-Hacohen R. Duridanova D. Meiri H. Rahamimoff R. (2004). Hydrogen ions control synaptic vesicle ion channel activity in torpedo electromotor neurones. J. Physiol. 556, 347–352. doi: 10.1113/jphysiol.2003.058818, PMID: 14978200 PMC1664946

[ref2] Arraud N. Linares R. Tan S. Gounou C. Pasquet J. M. Mornet S.. (2014). Extracellular vesicles from blood plasma: determination of their morphology, size, phenotype and concentration. J. Thromb. Haemost. 12, 614–627. doi: 10.1111/jth.12554, PMID: 24618123

[ref3] Bacia K. Majoul I. V. Schwille P. (2002). Probing the endocytic pathway in live cells using dual-color fluorescence cross-correlation analysis. Biophys. J. 83, 1184–1193. doi: 10.1016/S0006-3495(02)75242-9, PMID: 12124298 PMC1302220

[ref4] Bowen M. E. Weninger K. Brunger A. T. Chu S. (2004). Single molecule observation of liposome-bilayer fusion thermally induced by soluble N-ethyl Maleimide sensitive-factor attachment protein receptors (SNAREs). Biophys. J. 87, 3569–3584. doi: 10.1529/biophysj.104.048637, PMID: 15347585 PMC1304822

[ref5] Burré J. (2015). The synaptic function of α-synuclein. J. Parkinsons Dis. 5, 699–713. doi: 10.3233/JPD-150642, PMID: 26407041 PMC4927875

[ref6] Calì T. Ottolini D. Brini M. (2014). Calcium signaling in Parkinson’s disease. Cell Tissue Res. 357, 439–454. doi: 10.1007/s00441-014-1866-0 24781149

[ref7] Chan Y.-H. M. van Lengerich B. Boxer S. G. (2009). Effects of linker sequences on vesicle fusion mediated by lipid-anchored DNA oligonucleotides. Proc. Natl. Acad. Sci. U. S. A. 106, 979–984. doi: 10.1073/pnas.0812356106, PMID: 19164559 PMC2633564

[ref8] Cornell C. E. Mileant A. Thakkar N. Lee K. K. Keller S. L. (2020). Direct imaging of liquid domains in membranes by cryo-electron tomography. Proc. Natl. Acad. Sci. 117, 19713–19719. doi: 10.1073/pnas.2002245117, PMID: 32759217 PMC7443872

[ref10] Cypionka A. Stein A. Hernandez J. M. Hippchen H. Jahn R. Walla P. J. (2009). Discrimination between docking and fusion of liposomes reconstituted with neuronal SNARE-proteins using FCS. Proc. Natl. Acad. Sci. U. S. A. 106, 18575–18580. doi: 10.1073/pnas.0906677106, PMID: 19843696 PMC2764736

[ref11] Davidson W. Jonas A. Clayton D. George J. (1998). Stabilization of alpha-Synuclein secondary structure upon binding to synthetic membranes. J. Biol. Chem. 273, 9443–9449. doi: 10.1074/jbc.273.16.9443, PMID: 9545270

[ref12] Diao J. Ishitsuka Y. Lee H. Joo C. Su Z. Syed S.. (2012). A single vesicle-vesicle fusion assay for in vitro studies of snares and accessory proteins. Nat. Protoc. 7, 921–934. doi: 10.1038/nprot.2012.020, PMID: 22582418 PMC4410872

[ref13] Emelyanov A. Shtam T. Kamyshinsky R. Garaeva L. Verlov N. Miliukhina I.. (2020). Cryo-electron microscopy of extracellular vesicles from cerebrospinal fluid. PLoS One 15, –e0227949. doi: 10.1371/journal.pone.0227949, PMID: 31999742 PMC6991974

[ref14] Fahn S. (2008). The history of dopamine and levodopa in the treatment of Parkinson’s disease. Mov. Disord. 23, S497–S508. doi: 10.1002/mds.22028 18781671

[ref15] Fang Z. Patterson B. R. Turner M. E. (1993). Modeling particle size distributions by the Weibull distribution function. Mater. Charact. 31, 177–182. doi: 10.1016/1044-5803(93)90058-4

[ref16] Février B. Raposo G. (2004). Exosomes: endosomal-derived vesicles shipping extracellular messages. Curr. Opin. Cell Biol. 16, 415–421. doi: 10.1016/j.ceb.2004.06.003, PMID: 15261674

[ref17] Finger F. P. White J. G. (2002). Fusion and fission: membrane trafficking in animal cytokinesis. Cells 108, 727–730. doi: 10.1016/S0092-8674(02)00668-2 11955425

[ref18] Fix M. Melia T. J. Jaiswal J. K. Rappoport J. Z. You D. Sollner T. H.. (2004). Imaging single membrane fusion events mediated by SNARE proteins. Proc. Natl. Acad. Sci. U. S. A. 101, 7311–7316. doi: 10.1073/pnas.0401779101, PMID: 15123811 PMC409915

[ref19] Flagmeier P. De S. Wirthensohn D. C. Lee S. F. Vincke C. Muyldermans S.. (2017). Ultrasensitive measurement of ca 2+ influx into lipid vesicles induced by protein aggregates. Angew. Chemie Int. Ed. 56, 7750–7754. doi: 10.1002/anie.201700966, PMID: 28474754 PMC5615231

[ref20] Fouke K. E. Wegman M. E. Weber S. A. Brady E. B. Román-Vendrell C. Morgan J. R. (2021). Synuclein regulates synaptic vesicle clustering and docking at a vertebrate synapse. Front. Cell Dev. Biol. 9:774650. doi: 10.3389/fcell.2021.774650, PMID: 34901020 PMC8660973

[ref21] Furt F. Moreau P. (2009). Importance of lipid metabolism for intracellular and mitochondrial membrane fusion/fission processes. Int. J. Biochem. Cell Biol. 41, 1828–1836. doi: 10.1016/j.biocel.2009.02.005, PMID: 19703652

[ref22] Fusco G. Pape T. Stephens A. D. Mahou P. Costa A. R. Kaminski C. F.. (2016). Structural basis of synaptic vesicle assembly promoted by α-synuclein. Nat. Commun. 7:12563. doi: 10.1038/ncomms12563, PMID: 27640673 PMC5031799

[ref23] Gaspar R. Idini I. Carlström G. Linse S. Sparr E. (2021). Transient lipid-protein structures and selective ganglioside uptake during α-Synuclein-lipid co-aggregation. Front. Cell Dev. Biol. 9:622764. doi: 10.3389/fcell.2021.622764, PMID: 33681202 PMC7930334

[ref24] Grey M. Linse S. Nilsson H. Brundin P. Sparr E. (2011). Membrane interaction of α-synuclein in different aggregation states. J. Parkinsons Dis. 1, 359–371. doi: 10.3233/JPD-2011-11067, PMID: 23933657

[ref25] Hallett F. R. Watton J. Krygsman P. (1991). Vesicle sizing: number distributions by dynamic light scattering. Biophys. J. 59, 357–362. doi: 10.1016/S0006-3495(91)82229-9, PMID: 19431789 PMC1281152

[ref26] Han J. Pluhackova K. Böckmann R. A. (2017). The multifaceted role of SNARE proteins in membrane fusion. Front. Physiol. 8:5. doi: 10.3389/fphys.2017.00005, PMID: 28163686 PMC5247469

[ref27] Hannestad J. K. Rocha S. Agnarsson B. Zhdanov V. P. Wittung-Stafshede P. Höök F. (2020). Single-vesicle imaging reveals lipid-selective and stepwise membrane disruption by monomeric α-synuclein. Proc. Natl. Acad. Sci. U. S. A. 117, 14178–14186. doi: 10.1073/pnas.1914670117, PMID: 32513706 PMC7322013

[ref28] Hatzakis N. S. Bhatia V. K. Larsen J. Madsen K. L. Bolinger P.-Y. Kunding A. H.. (2009). How curved membranes recruit amphipathic helices and protein anchoring motifs. Nat. Chem. Biol. 5, 835–841. doi: 10.1038/nchembio.213, PMID: 19749743

[ref29] Heberle F. A. Doktorova M. Scott H. L. Skinkle A. D. Waxham M. N. Levental I. (2020). Direct label-free imaging of nanodomains in biomimetic and biological membranes by cryogenic electron microscopy. Proc. Natl. Acad. Sci. U. S. A. 117, 19943–19952. doi: 10.1073/pnas.2002200117, PMID: 32759206 PMC7443941

[ref30] Hellstrand E. Grey M. Ainalem M.-L. Ankner J. Forsyth V. T. Fragneto G.. (2013). Adsorption of α-synuclein to supported lipid bilayers: positioning and role of electrostatics. ACS Chem. Neurosci. 4, 1339–1351. doi: 10.1021/cn400066t, PMID: 23823878 PMC3798988

[ref31] Höög J. L. Lötvall J. (2015). Diversity of extracellular vesicles in human ejaculates revealed by cryo-electron microscopy. J. Extracell. Vesicles 4:28680. doi: 10.3402/jev.v4.28680, PMID: 26563734 PMC4643196

[ref32] Jähnig F. (1984). Lipid exchange between membranes. Biophys. J. 46, 687–694. doi: 10.1016/S0006-3495(84)84067-9, PMID: 6518251 PMC1435098

[ref33] Jo E. McLaurin J. A. Yip C. M. St. George-Hyslop P. Fraser P. E. (2000). α-Synuclein membrane interactions and lipid specificity. J. Biol. Chem. 275, 34328–34334. doi: 10.1074/jbc.M004345200, PMID: 10915790

[ref34] Kim S. Seo J. H. Suh Y. H. (2004). α-Synuclein, Parkinson’s disease, and Alzheimer’s disease. Park. Relat. Disord. 10, S9–S13. doi: 10.1016/j.parkreldis.2003.11.005 15109581

[ref35] Kinoshita M. Arai H. Fukasawa M. Watanabe T. Tsukamoto K. Hashimoto Y.. (1993). Apolipoprotein E enhances lipid exchange between lipoproteins mediated by cholesteryl ester transfer protein. J. Lipid Res. 34, 261–268. doi: 10.1016/S0022-2275(20)40753-9, PMID: 8429260

[ref36] Kunding A. H. Mortensen M. W. Christensen S. M. Stamou D. (2008). A fluorescence-based technique to construct size distributions from single-object measurements: application to the extrusion of lipid vesicles. Biophys. J. 95, 1176–1188. doi: 10.1529/biophysj.108.128819, PMID: 18424503 PMC2479610

[ref37] Lautenschläger J. Stephens A. D. Fusco G. Ströhl F. Curry N. Zacharopoulou M.. (2018). C-terminal calcium binding of α-synuclein modulates synaptic vesicle interaction. Nat. Commun. 9:712. doi: 10.1038/s41467-018-03111-4, PMID: 29459792 PMC5818535

[ref38] Lee J. Y. Kim H. S. (2017). Extracellular vesicles in neurodegenerative diseases: a double-edged sword. Tissue Eng. Regen. Med. 14, 667–678. doi: 10.1007/s13770-017-0090-x, PMID: 30603519 PMC6171665

[ref39] LewisP. A. (2021). Vesicular dysfunction and pathways to neurodegeneration. Essays Biochem. 65, 941–948. doi: 10.1042/EBC20210034, PMID: 34897416

[ref40] Lipp N.-F. Ikhlef S. Milanini J. Drin G. (2020). Lipid exchangers: cellular functions and mechanistic links with phosphoinositide metabolism. Front. Cell Dev. Biol. 8:663. doi: 10.3389/fcell.2020.00663, PMID: 32793602 PMC7385082

[ref41] Liu T. Tucker W. C. Bhalla A. Chapman E. R. Weisshaar J. C. (2005). SNARE-driven, 25-millisecond vesicle fusion in vitro. Biophys. J. 89, 2458–2472. doi: 10.1529/biophysj.105.062539, PMID: 16055544 PMC1366745

[ref42] Lou X. Kim J. Hawk B. J. Shin Y.-K. (2017). α-Synuclein may cross-bridge v-SNARE and acidic phospholipids to facilitate SNARE-dependent vesicle docking. Biochem. J. 474, 2039–2049. doi: 10.1042/BCJ20170200, PMID: 28495859 PMC5772654

[ref43] Makasewicz K. Wennmalm S. Linse S. Sparr E. (2022). α-Synuclein-induced deformation of small Unilamellar vesicles. QRB Discov. 3, e10, 1–9. doi: 10.1017/qrd.2022.9 PMC10392696 37529290

[ref44] Makasewicz K. Wennmalm S. Stenqvist B. Fornasier M. Andersson A. Jönsson P.. (2021). Cooperativity of α-Synuclein binding to lipid membranes. ACS Chem. Neurosci. 12, 2099–2109. doi: 10.1021/acschemneuro.1c00006, PMID: 34076426 PMC8291482

[ref45] Man W. K. De Simone A. Barritt J. D. Vendruscolo M. Dobson C. M. Fusco G. (2020). A role of cholesterol in modulating the binding of α-Synuclein to synaptic-like vesicles. Front. Neurosci. 14:18. doi: 10.3389/fnins.2020.00018, PMID: 32063829 PMC7000551

[ref46] Pencer J. Hallett F. R. (2003). Effects of vesicle size and shape on static and dynamic light scattering measurements. Langmuir 19, 7488–7497. doi: 10.1021/la0345439

[ref47] Peretti D. Kim S. Tufi R. Lev S. (2020). Lipid transfer proteins and membrane contact sites in human cancer. Front. Cell Dev. Biol. 7:371. doi: 10.3389/fcell.2019.00371, PMID: 32039198 PMC6989408

[ref48] Raposo G. Stoorvogel W. (2013). Extracellular vesicles: exosomes, microvesicles, and friends. J. Cell Biol. 200, 373–383. doi: 10.1083/jcb.201211138, PMID: 23420871 PMC3575529

[ref49] Sezgin E. (2017). Super-resolution optical microscopy for studying membrane structure and dynamics. J. Phys. Condens. Matter 29:273001. doi: 10.1088/1361-648X/aa7185, PMID: 28481213 PMC5952331

[ref50] Simonsson L. Jönsson P. Stengel G. Höök F. (2010). Site-specific DNA-controlled fusion of single lipid vesicles to supported lipid bilayers. Chem. Phys. Chem. 11, 1011–1017. doi: 10.1002/cphc.200901010, PMID: 20301177

[ref51] Südhof T. C. (1995). The synaptic vesicle cycle: a cascade of protein–protein interactions. Nature 375, 645–653. doi: 10.1038/375645a0, PMID: 7791897

[ref52] Thorsteinsson K. Olsén E. Schmidt E. Pace H. Bally M. (2020). FRET-based assay for the quantification of extracellular vesicles and other vesicles of complex composition. Anal. Chem. 92, 15336–15343. doi: 10.1021/acs.analchem.0c02271, PMID: 33179908 PMC7735656

[ref53] Vitale N. Gasman S. Caumont A.-S. Gensse M. Galas M.-C. Chasserot-Golaz S.. (2000). Insight in the exocytotic process in chromaffin cells: regulation by trimeric and monomeric G proteins. Biochimie 82, 365–373. doi: 10.1016/S0300-9084(00)00198-X, PMID: 10865124

[ref54] Yoon T.-Y. Okumus B. Zhang F. Shin Y.-K. Ha T. (2006). Multiple intermediates in SNARE-induced membrane fusion. Proc. Natl. Acad. Sci. U. S. A. 103, 19731–19736. doi: 10.1073/pnas.0606032103, PMID: 17167056 PMC1698870

[ref55] Yuana Y. Koning R. I. Kuil M. E. Rensen P. C. N. Koster A. J. Bertina R. M.. (2013). Cryo-electron microscopy of extracellular vesicles in fresh plasma. J. Extracell. Vesicles 2:21494. doi: 10.3402/jev.v2i0.21494, PMID: 24455109 PMC3895263

[ref56] Zabeo D. Cvjetkovic A. Lässer C. Schorb M. Lötvall J. Höög J. L. (2017). Exosomes purified from a single cell type have diverse morphology. J. Extracell. Vesicles 6:1329476. doi: 10.1080/20013078.2017.1329476, PMID: 28717422 PMC5505001

